# Effects of antipsychotics on human cognitive function: causal evidence from healthy volunteers following sustained D2/D3 antagonism, D2/D3 partial agonism and placebo

**DOI:** 10.1038/s41380-025-03116-8

**Published:** 2025-07-19

**Authors:** Martin Osugo, Uzma Zahid, Pierluigi Selvaggi, Alexandria Chilimidos, Valeria Finelli, George E. Chapman, Thomas Whitehurst, Ellis Chika Onwordi, Robin M. Murray, Matthew B. Wall, Tiago Reis Marques, Mitul A. Mehta, Oliver D. Howes

**Affiliations:** 1 Department of Psychosis Studies, Institute of Psychiatry, Psychology & Neuroscience, King’s College London, London, UK; 2 Psychiatric Imaging Group, Medical Research Council, London Institute of Medical Sciences, Hammersmith Hospital, London, UK; 3 South London and Maudsley NHS Foundation Trust, London, UK; 4 Department of Psychology, Institute of Psychiatry, Psychology & Neuroscience, King’s College London, London, UK; 5 Department of Neuroimaging, Institute of Psychiatry, Psychology and Neuroscience, King’s College London, London, UK; 6 Department of Translational Biomedicine and Neuroscience, University of Bari Aldo Moro, Bari, Italy; 7 Division of Psychiatry, Faculty of Brain Sciences, University College London, London, UK; 8 North London NHS Foundation Trust, London, UK; 9 East London NHS Foundation Trust, London, UK; 10 Institute of Clinical Sciences (ICS), Faculty of Medicine, Imperial College London, London, UK; 11 Centre for Psychiatry and Mental Health, Wolfson Institute of Population Health, Queen Mary University of London, London, UK; 12 Perceptive, London, UK; 13 Faculty of Medicine, Imperial College London, London, UK

**Keywords:** Schizophrenia, Neuroscience, Molecular biology, Psychology

## Abstract

Dopamine D2/D3 receptor modulation with antipsychotics is thought to affect cognitive function, but causal evidence in humans is scant, and largely limited to single administrations. Clarifying this is of importance given the widespread use of antipsychotics, and to understand the role of D2/D3 signalling in human cognition. We therefore conducted a double-blind, placebo-controlled crossover study following sustained administration of either a dopamine D2/D3 receptor antagonist (amisulpride at 400 mg daily) or a D2/D3 partial agonist (aripiprazole at 10 mg daily) to two separate samples of healthy humans (total n = 50) for 7 days per condition. We assessed cognitive function using a computerised visuospatial working memory (VS-WM) task, and sustained attention and response inhibition using the Sustained Attention to Response Task (SART). We found that both amisulpride and aripiprazole caused impairments in VS-WM function compared to placebo on the Balanced Integration Score (amisulpride: p = 0.0079; aripiprazole: p = 0.015). Both antipsychotics impaired VS-WM performance in terms of response latency (amisulpride: p = 5.5 × 10^−7^; aripiprazole: p = 0.022), but did not affect response accuracy. Response latency deficits were not correlated with motor impairments induced by either drug, and we also found no effect of either drug on the SART measures, or on subjective alertness, suggesting that D2/D3 antagonism or partial agonism did not cause a generalised cognitive or motor deficit but specifically impaired cognition during VS-WM. This study provides the first causal evidence in healthy humans that working memory function is impaired following either sustained antagonism or partial agonism of D2/D3 receptors by antipsychotic drugs.

## Introduction

Several lines of evidence show that dopaminergic signalling is critical for cognitive functioning, particularly for executive functions such as working memory (WM), attention, and response inhibition [1, 2]. For example, in monkeys, depletion of prefrontal cortex (PFC) dopamine produces deficits in visuospatial delayed WM as severe as that caused by surgical ablation of the PFC, and microdialysis studies show that WM tasks lead to dopamine release in the PFC of monkeys and rats [3–5]. Furthermore, D2/D3 receptor modulation has been shown to regulate sustained attention in rodents, whilst mice lacking D2/D3 receptors show impairments in response inhibition and in spatial WM, with increasing impairments at increasing delays [6–9].

In healthy humans, Positron Emission Tomography (PET) studies show that sustained attention, response inhibition and WM tasks are associated with dopamine release, and striatal D2/D3 receptor availability [10–12]. Cognitive impairments are also a core feature of neuropsychiatric disorders associated with dopamine dysfunction, such as schizophrenia, Parkinson’s disease, and Huntington’s disease, and PET imaging shows an association between D2/D3 receptor availability or dopamine release and WM impairments in these disorders [13–17].

From the advent of pharmacological treatments for psychosis over 70 years ago until very recently, all available antipsychotic drugs blocked D2/D3 receptors [18, 19]. Observational studies in chronic schizophrenia and in dementia suggest that they may therefore exacerbate pre-existing cognitive deficits, including in WM and attention [20–22]. Moreover, the subjective experience of cognitive impairment after chronic antipsychotic use is commonly reported by patients, and may lead to treatment discontinuation [23]. Contrastingly, meta-analyses of placebo-controlled Randomised Control Trials (RCTs) in these disorders show that antipsychotics do not differ from placebo in their effects on cognition [24, 25]. However, disentangling changes in cognition from behavioural changes is challenging in these trials as they are largely conducted in patients experiencing acute psychotic or behavioural exacerbations [24, 26]. Clarifying the cognitive effects of antipsychotics is an important question, as they are prescribed long-term to tens of millions of people worldwide each year [27, 28].

Several studies have investigated a causal role for D2/D3 receptors in regulating human cognition by administering pharmacological challenges to healthy volunteers in placebo-controlled designs. These show that single doses of D2/D3 antagonists impair response inhibition in healthy humans [29, 30], although studies investigating sustained attention have shown both impairments and no effect relative to placebo following acute D2/D3 blockade [30–34]. Findings on WM are also inconsistent, with some studies finding that acute D2/D3 antagonism impairs WM performance, whilst others demonstrate no effect or improvements in performance compared to placebo [29, 35–41].

A potential explanation for the discrepancy between the subjective experience of patients who take long term antipsychotics, and these mixed findings from single dose studies in healthy volunteers, is that cognitive impairments increase after repeated antipsychotic administration. Supporting this, in rodents, impairments in recognition memory induced by D2/D3 blockade have been shown to worsen with repeated administration [42]. So far, the longest double-blind, placebo-controlled studies of cognitive function following antipsychotic administration in healthy humans found impairments in sustained attention following D2/D3 antagonism for 4–5 days [34, 43]. In these studies, cognitive impairments were more pronounced following repeated dosing compared to single dosing for amisulpride 400 mg daily and haloperidol 3–4 mg daily, but not olanzapine 3 mg daily [34, 43]. However, in addition to relatively small sample sizes (n ≤ 15), they did not assess WM or response inhibition [34, 43, 44]. A single-blind investigation in healthy humans also found impairments in processing speed and reaction time compared to placebo in tests of attentional performance, following administration of D2/D3 modulators for 7 days, but found no effect on WM accuracy using a relatively simple pen and paper WM task [45]. This task could not measure WM response latency, which has been shown to be modulated by acute D2/D3 antagonism [36]. Moreover, in addition to participants not being blinded, the study did not report separate effects by drug mechanism, pooling the effects of the D2/D3 antagonist haloperidol with the D2/D3 partial agonist aripiprazole and reserpine, which depletes synaptic dopamine (therefore affecting neurotransmission at all dopamine receptor subtypes) in addition to depleting synaptic serotonin and noradrenaline [45]. This is important as an additional question is whether partial agonism also impairs cognitive function. One prior study in rats found that administration of the D2/D3 antagonist risperidone resulted in poorer spatial WM performance compared to administration of the D2/D3 partial agonist aripiprazole [46]. Moreover, single doses of full dopamine agonists have been shown to enhance WM, or to have no effect in humans [35, 47–49]. However, in healthy humans, the effects of sustained D2/D3 partial agonists on cognition have not been tested despite the hypothesis that D2/D3 partial agonists are less cognitively impairing compared to full antagonists, and their widespread use [46, 50, 51].

In this study we therefore aimed to test the effect of a selective D2/D3 antagonist antipsychotic and a D2/D3 partial agonist antipsychotic on human cognitive function, following sustained administration to healthy humans in a double-blind, placebo-controlled crossover study. This design allowed us to disentangle the effects of disease state from drug effects, whilst multiple dosing is a more suitable translational model of drug effects, as discussed above. Based on the preclinical and acute studies above, we hypothesised that, compared to placebo, the D2/D3 receptor antagonist amisulpride would impair sustained attention, response inhibition and WM, whilst the sustained D2/D3 partial agonist aripiprazole would cause less impairment than full D2/D3 antagonism.

## Methods

### Ethical approval

This study was approved by the London - West London and GTAC NHS Research Ethics Committee (Ethics Committee Reference Number: 18/LO/1044). All subjects provided written, informed consent prior to participation. The study was conducted in accordance with the Declaration of Helsinki.

### Participants

Healthy volunteers aged 18–65 years were recruited by public advertisement. Exclusion criteria were; history of psychiatric illness (including alcohol/substance dependence or abuse, other than caffeine/nicotine) as determined by self-report and the Mini-International Neuropsychiatric Interview, current use of any illicit substances as determined by urine drug of abuse testing and self-report, pregnancy as determined by urine pregnancy testing and self-report, self-report of a first degree relative with a psychotic disorder, current or significant previous use of psychotropic or dopamine modulating drugs, breastfeeding, or participation in a study of unlicensed medicines within the previous 30 days, self-report or clinical findings of significant CNS disorder (e.g. significant head trauma, epilepsy etc.), significant medical disorder, contraindications to dopamine antagonists/partial agonists or MRI scanning, or clinically relevant abnormal findings at the screening assessment, as determined by the principal investigator.

### Study design

**Fig. 1:**
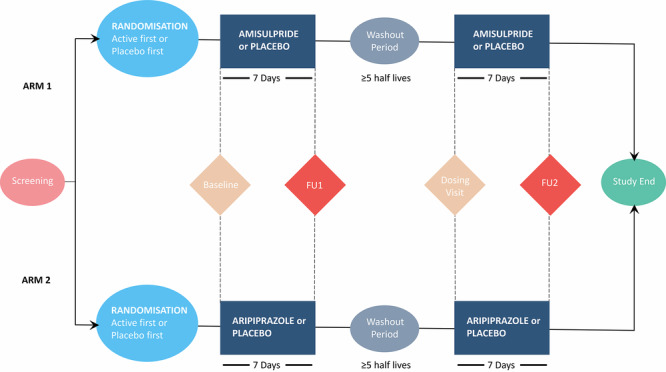
The order of study interventions and measures for the two arms of the study is shown. The arms were conducted sequentially, with Arm 1 completed prior to Arm 2 commencing. The order of treatments within each arm (active drug or placebo first) was randomised and counter-balanced to ensure approximately equal numbers of subjects receiving drug or placebo first. Subjects and investigators were blind to the treatment allocation. The washout period was a minimum 10 days for amisulpride, and a minimum of 28 days for aripiprazole. Cognitive testing and assessment of parkinsonian symptoms using the Simpson Angus Scale (SAS) were performed at baseline, follow up (FU) 1 and FU 2 visits.

This was a single-centre, double-blind, placebo-controlled, crossover study (Fig. 1). Two independent groups of healthy volunteers received either amisulpride and placebo or aripiprazole and placebo for seven days each. The amisulpride and placebo crossover study (arm 1) and aripiprazole and placebo crossover study (arm 2) were conducted sequentially at the same site. The order of administration within each arm was randomised and counter-balanced to ensure approximately equal numbers received placebo or active drug first. Amisulpride doses were titrated up to 400 mg/day (Day 1:200 mg, Day 2:300 mg, Days 3–7: 400 mg). Aripiprazole doses were titrated up to 10 mg/day (Day 1:5 mg, Day 2:5 mg, Days 3–7:10 mg). The doses used for each drug are considered the minimum clinically effective doses in the treatment of schizophrenia by the Maudsley Prescribing Guidelines [52].

Volunteers were evaluated at a screening appointment prior to randomisation. After the screening visit, eligible subjects were randomised to treatment order (amisulpride or placebo first in arm 1, aripiprazole or placebo first in arm 2). These participants subsequently returned for the baseline assessment, following which the first dose of study medication was administered at the research facility, and the remaining six days of medications were dispensed to be taken at home. After completing the first treatment period, participants returned for outcome and safety assessment after seven days, before entering a washout period of at least five half-lives of the drug and its active metabolites (minimum 10 days for amisulpride, minimum 28 days for aripiprazole). After the washout period, participants returned to the research facility and commenced the other treatment condition. Compliance was assessed at the end of each treatment week with pill counts and blood sampling for testing of drug levels.

### Outcome measures

Demographic information was collected at the screening appointment by self-report. Cognitive tests, subjective state, and parkinsonian symptoms (Simpson Angus Scale (SAS)) were assessed at baseline, and repeated following one week of amisulpride or aripiprazole, and following one week of placebo. Plasma amisulpride or aripiprazole + de-hydroaripiprazole levels were measured following each treatment week, and aripiprazole + de-hydroaripiprazole levels were also measured following the washout period to detect and exclude slow metabolisers of aripiprazole. Detection was by selective reaction monitoring using tandem mass-spectrometry. Instrument control was via a PC using the Agilent EZChrom, and Thermo Xcalibur software; data acquisition and processing was via the Thermo Xcalibur software.

### Cognitive tests and subjective measures

The tasks were presented on a laptop computer using the Gorilla Experiment Builder [53]. Data were collected between 19^th^ February 2019 and 5^th^ April 2023. Visuospatial working memory (VS-WM) and Visual Analogue Scale (VAS) responses were recorded using a standard wired mouse.

Subjective state was assessed using the Bond and Lader VAS, which consists of 16 items. Subjects indicate their current subjective state on each item on a dimensional scale ranging from 1–100 [54]. VAS data were collected using a laptop computer immediately prior to cognitive testing.

We used the Sustained Attention to Response Task (SART) to measure response inhibition and sustained attention [55]. The SART is a go/no-go task consisting of 225 trials, lasting approximately 4.3 min in total. Single digits between 1–9 are presented visually to participants for 900 ms each in random order, with each digit followed by a fixation cross presented for 250 ms. Participants are instructed to press the space bar for every digit except “3”. Each digit is presented 25 times, leading to 200 go trials and 25 no go trials. We extracted data on errors of commission (responding when the number 3 is presented), errors of omission (failing to respond to a non 3 digits within 900 ms), total errors (errors commission + errors omission) and mean reaction time on correct trials.

**Fig. 2:**
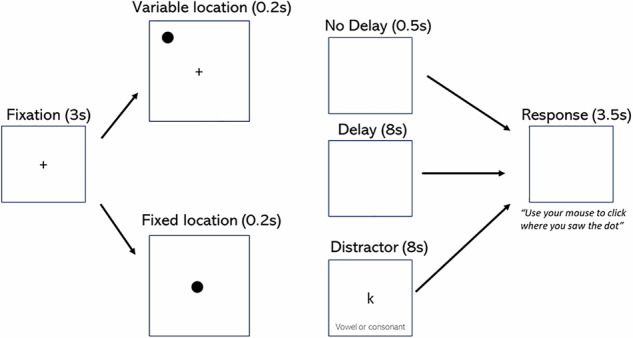
The computerised visuospatial working memory task used in the study is illustrated. In each trial, a central fixation cross was presented for 3.0 s, before the target (a black circle) appeared for 0.2 s. The task consisted of a total of 66 trials of varying difficulty, presented in random order. Trial difficulty was varied by altering the target location, by increasing the delay between target presentation and response, and by presenting a distractor task between target presentation and response. In fixed location trials, the target appeared in the centre of the screen, and in variable location trials the target appeared displaced from the central fixation cross. The three delay conditions between target presentation and response were: 1)0.5 s delay, 2)8 s delay, 3)8 s delay followed by a distractor task (vowel-consonant decision). Following each delay/distractor, participants were asked to recall the location of the target on a blank screen, presented for 3.5 s.

To measure VS-WM, we used a delayed response task adapted from previous tasks used with monkeys and humans, which has been shown to be dependent on PFC dopamine function [36, 56, 57]. (Fig. 2).

First, a central fixation cross (1.5 cm × 1.5 cm) appeared on the monitor for 3 seconds. Following this, the target (a black circle, 0.5 cm×0.5 cm) appeared on the screen for 0.2 s. In fixed location trials, the dot appeared in the centre of the screen, replacing the fixation cross. In variable location trials, the dot appeared displaced from the fixation cross in one of 48 possible locations. There were then three different delay types prior to the response. In the no delay condition, the response screen appeared 0.5 s after the presentation of the target, with a blank screen intervening for this period. In the delay condition, the blank screen appeared for 8 s after the presentation of the target. In the distractor condition, a letter decision task followed the 8 s delay. In this, the participant had to decide whether a letter is a vowel or a consonant and the task did not progress until a response was recorded. Following the no delay, delay or distractor conditions, the participants were asked to use the mouse to click where they saw the dot on a blank screen, with no fixation cross. They had to respond within 3.5 s in order for the response to be recorded. The task consisted of a total of 66 trials, with 22 trials in each delay condition (16 variable location trials and 6 fixed location trials). The order of trials was randomised. For the analysis of VS-WM, we extracted data from dot location trials on response error (calculated as the Euclidean distance between the response and the target), and reaction time. Only first responses were considered. As a control for motor slowing, we also extracted data on response time in all vowel-consonant distractor trials (as this had the same response requirements, but did not require VS-WM).

### Statistical analysis

Data were analysed in Matlab (version 9.13.0.2049777, The MathWorks Inc; Massachusetts, USA) and SPSS (v25, IBM Corp; New York, USA).

Comparisons between demographic variables for the amisulpride and aripiprazole samples were performed using two sided independent-sample t-tests or Mann-Whitney U tests for continuous variables, and chi-squared tests for categorical variables. Mann-Whitney U tests were used if the assumptions of normality (from the Shapiro-Wilks test) or equal variances (from Levene’s test) were violated.

For both tasks, we excluded sessions where participants failed to respond in >25% of trials where a response was required, in line with previous analyses [58]. After exclusion of these sessions and subjects with undetectable plasma drug levels following the active treatment week, we analysed the data for all eligible subjects who completed at least one post baseline treatment condition with usable data.

In previous studies of WM and sustained attention tasks where impairments have been demonstrated following single doses of D2/D3 antagonists, they have been seen in response accuracy, response latency or in both measures [30, 32, 35, 36, 38, 41, 43]. We therefore lacked a clear hypothesis as to whether D2/D3 modulation would primarily affect response latency or accuracy, and so our primary outcome measure for both tasks was the Balanced Integration Score (BIS), a single outcome measure which combines speed and accuracy to control for speed-accuracy trade-offs [59]. As recommended, we calculated the BIS by computing Z-scores for accuracy and reaction time, and then subtracting the Z-score for reaction time from the Z-score for accuracy [59]. The mean and sample standard deviation used to compute Z-scores were calculated over all cells that contributed variance (baseline, active drug and placebo sessions) [59]. For the VS-WM, accuracy Z-scores were multiplied by −1 so that a more negative BIS would reflect poorer performance, as is convention for this measure [59]. We then went on to conduct additional analyses of accuracy and reaction time data for both tasks. There are no prior double-blind studies on the effects of antipsychotics on sustained attention, response inhibition or VS-WM following 7 days of administration in healthy volunteers, but administration of amisulpride 400 mg for 5 days in healthy volunteers has been shown to result in impairments in sustained attention compared to placebo, with an effect size of approximately d = 0.77 [34]. We would require at least n = 24 participants in each drug condition to detect an effect of this magnitude, using a two-sided paired t-test with alpha=0.05 and power=0.95.

Task performance was analysed by fitting a generalised linear mixed-effects model in Matlab using the function fitglme, fit by Restricted Maximum Likelihood (REML) [60]. For the SART, the main predictor was the fixed effect of treatment condition (active drug or placebo). We also included random intercepts for each participant, by participant random slopes for the effect of treatment condition, and the fixed effects of baseline levels of the outcome variable of interest and treatment order (drug or placebo first). Errors were modelled with a Poisson distribution, as they are count data [61].

For the VS-WM task, the main predictor was the fixed effect of treatment condition (drug or placebo). We also included the fixed effects of baseline levels of the outcome variable of interest, treatment order (drug or placebo first), delay type (no delay, delay and distractor) and trial difficulty (fixed location vs variable location). We included exploratory fixed effects examining the interaction between treatment condition and delay, and the interaction between treatment condition and trial difficulty. We included random intercepts for each participant, and by-participant random slopes for the effect of treatment condition, delay type, and trial difficulty and the interaction between treatment condition*delay and treatment condition*trial difficulty [60].

We conducted *post hoc* tests on coefficients of the linear models using an F-test (coefTest in MATLAB) to investigate the significance of coefficients and to explore significant interactions, with multiple comparisons corrected for using FDR correction with the Benjamini-Hochberg procedure [62]. We extracted estimated marginal means and their standard errors from the general linear mixed effects models using the MATLB function emmeans, and plotted these in GraphPad Prism (Version 10.2.3, GraphPad Software; Massachusetts, USA).

We compared amisulpride-placebo differences to aripiprazole-placebo differences using two-sided Student’s t-tests to compare differences in regression slopes [63]. For the VS-WM task, we ran linear mixed models excluding interaction terms in order to generate effect sizes for this analysis.

For the VAS, we calculated two-factors representing alertness and tranquillity, as previously described [64]. Values from each of these factors were then input into a linear mixed model. Code for all linear mixed models are reported in the supplementary materials. Correlations between measures were analysed using Pearson’s correlations, or Spearman’s rank correlations if data were not normally distributed as determined by the Shapiro–Wilk test.

## Results

### Sample characteristics and demographics

Seventy-six healthy volunteers were randomised and a total of fifty completed treatment with either amisulpride and placebo, or aripiprazole and placebo. For a full description of adverse events and dropouts please see our previous publications [65–68]. There were no significant differences between the amisulpride (n = 25) and aripiprazole (n = 25) samples on any demographic variables or on proportion receiving placebo first. The sample characteristics have been previously published and are further described in Table 1 [65–67].

**Table 1:** Description of sample.

	Amisulpride (n = 25)	Aripiprazole (n = 25)	comparison
Sex	Female 15 (60%); Male 10 (40%)	Female 14 (56%); Male 11 (44%)	p = 0.78
Age (Years)	26.5 (7.6)	26.7 (8.7)	Mann-Whitney U: p = 0.82
Ethnicity	White 15, Asian 4, Black 1, Mixed/Other 5	White 16, Asian 5, Black 2, Mixed/Other 2	p = 0.62
BMI	23.6 (3.8)	22.4 (3.5)	2 sample t-test p = 0.25
Education	Postgraduate 6, Undergraduate 13, High School 4, Professional Qualification 1	Postgraduate 9, Undergraduate 6, High School 9, Other 1	p = 0.15
Employment	Student 19, Employed 6	Student 16, Employed 9	p = 0.36
Treatment order	Amisulpride first 13 (52%), placebo first 12 (48%)	Aripiprazole first 12 (48%), placebo first 13 (52%)	p = 0.78
Plasma levels (ug/L)	312.4 (SD 203.0). Range 29–719)	Aripiprazole 95.8 (SD 33.3). Range 35–174 Aripiprazole + De-hydroaripiprazole 126 (31.4). Range 58–193	N/A
Washout length (days)	23.6(13.7). Range 12–64	52.6 (37.6). Range 28–168	N/A
Time from last dose to cognitive testing (hours)	13.6 (4.1). Range 4.8–21.1	27.5 (3.5). Range 17.7–33.6	N/A

Values are mean (SD) for continuous variables, and frequencies for categorical variables unless otherwise stated.

Comparisons between demographic variables were performed using two-sided independent sample t-tests or Mann-Whitney U tests for continuous variables, and chi-squared tests for categorical variables.

### Effects of amisulpride and aripiprazole on SART

In the SART, there was no significant effect of amisulpride on the BIS, or on mean reaction time, errors of commission, errors of omission or total errors compared to placebo (Table 2). We also found no significant effect of aripiprazole compared to placebo on the BIS, mean reaction time, errors of commission, errors of omission or total errors in the SART (Table 2). Relative to placebo, amisulpride and aripiprazole did not differ in their effects on the SART (BIS: t(52) = −0.94, p = 0.35; response latency t(52) = −0.22, p = 0.83; total errors t(52) = 0.06, p = 0.95; errors of commission t(52) = −0.33, p = 0.74).

**Table 2:** Effects of amisulpride vs placebo and aripiprazole vs placebo on SART outcomes.

Amisulpride								
Outcome variable	Amisulpride estimated marginal mean (SEM)	Placebo estimated marginal mean (SEM)	Fixed effect of amisulpride (beta)	SE of beta value	p-value (amisulpride vs placebo)	Model R^2^		p-value (effect of order)
Balanced Integration Score	1.0 (0.3)	1.1(0.4)	−0.16	0.26	0.55	0.69		0.37
Reaction time	315.9 (10.1)	313.4 (8.5)	2.51	6.84	0.72	0.90		0.76
Errors commission	6.0 (1.1)	6.3 (1.2)	−0.043	0.12	0.72	0.56		0.79
Errors omission	1.1 (1.35)	0.6 (1.4)	0.54	0.34	0.12	0.52		0.40
Total errors	6.9 (1.1)	7.0 (1.2)	−0.0073	0.11	0.94	0.67		0.89
**Aripiprazole**								
Outcome variable	Aripiprazole estimated marginal mean (SEM)	Placebo estimated marginal mean (SEM)	Fixed effect of aripiprazole (beta)	SE of beta value	p-value (aripiprazole vs placebo)		Model R^2^	p-value (effect of order)
Balanced Integration Score	1.5 (0.3)	1.2 (0.4)	0.22	0.31	0.49		0.88	0.16
Reaction time	325.4 (13.6)	320.3 (8.5)	5.12	10.0	0.61		0.88	0.72
Errors commission	4.5 (1.2)	4.5 (1.2)	0.02	0.15	0.90		0.65	0.69
Errors omission	0.4 (1.5)	0.5 (1.6)	0.16	0.33	0.64		0.64	0.07
Total errors	5.1 (1.2)	5.2 (1.2)	−0.019	0.15	0.90		0.83	0.26

### Effects of amisulpride on VS-WM

**Fig. 3:**
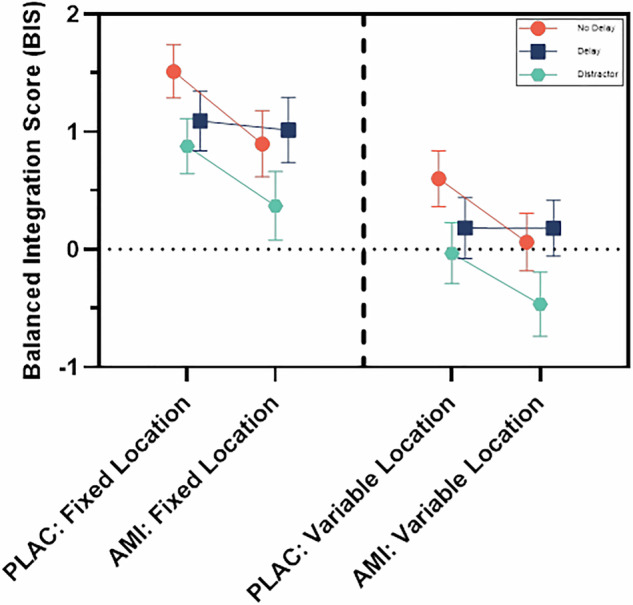
Balanced Integration Score (BIS) data from visuospatial working memory task, comparing placebo (PLAC) to amisulpride (AMI) in fixed location trials (left) and variable location trials (right), with separate coloured lines for delay conditions. The BIS measures accuracy adjusted for reaction time; lower scores indicate poorer performance. Values are estimated marginal means ± SEM from generalised linear mixed model. The main effect of treatment condition showed that amisulpride led to poorer performance compared to placebo. Analysis of task effects showed poorer performance in variable location trials compared to fixed location control trials, and poorer performance with increasing distraction/delay. There was no interaction between treatment condition and trial difficulty or treatment condition and delay. Model r^2^ = 0.84.

Analysis of the main effect of treatment condition showed that amisulpride significantly impaired performance compared to placebo on the BIS (p = 0.0079, F = 3.53, df1 = 4, df2 = 272) (Fig. 3). Analysis of task effects showed a significant main effect of trial difficulty on the BIS, with poorer performance in variable location compared to fixed location trials (p = 3.56 × 10^−7^, F = 15.69, df1 = 2, df2 = 272). There was also a significant main effect of delay on the BIS (p = 0.0009, F = 4.83, df1 = 4, df2 = 272]. *Post hoc* tests revealed that there was significantly poorer performance with increasing delay/distraction, with poorer performance in the delay condition compared to the no delay condition (FDR corrected p-value = 0.031), and significantly poorer performance in the distractor condition compared to the delay (FDR corrected p-value = 0.0076) and no delay conditions (FDR corrected p = 0.012). There was no effect of order (p = 0.27, F = 1.20, df1 = 1, df2 = 272).

There was no significant interaction between treatment condition and delay (p = 0.10, F = 2.30, df1 = 2, df2 = 272), or between treatment condition and trial difficulty (p = 0.71, F = 0.14, df1 = 1, df2 = 272), indicating that the main effect of amisulpride did not differ between variable location and fixed location trials, or between delay conditions.

Having shown that amisulpride impaired task performance on the BIS, we sought to determine whether this was due to impairments in response accuracy or response latency. We found that there was no main effect of treatment condition on VS-WM response accuracy (p = 0.95, F = 0.17, df1 = 4, df2 = 2917), but that there was a significant main effect of treatment condition on VS-WM response latency (p = 5.47 × 10^−7^, F = 8.71, df1 = 4, df2 = 2917), with slower responses on amisulpride compared to placebo (Supplementary Figures S1 & S2). However, we found no effect of amisulpride compared to placebo on response time to the vowel/consonant distractor during the task (b = −69.23, t(1030) = −0.68, p = 0.50).

As detailed in our previous publication, we found that amisulpride caused parkinsonian symptoms compared to placebo on the SAS [65]. We found that there was no relationship between the change in parkinsonian symptoms between the baseline assessment and the amisulpride assessment, or between the placebo assessment and the amisulpride assessment, and the difference in response latency in all trials across the same time periods (n = 23, rho = −0.32, p = 0.13 for amisulpride vs baseline; n = 20, rho = 0.07, p = 0.78 for amisulpride vs placebo). We also found no relationship between the change in the BIS or in response latency between the baseline and amisulpride assessments and plasma amisulpride levels (BIS: n = 24, rho = 0.02, p = 0.93. Response latency: n = 24, rho = 0.16, p = 0.49).

### Effects of aripiprazole on VS-WM

**Fig. 4:**
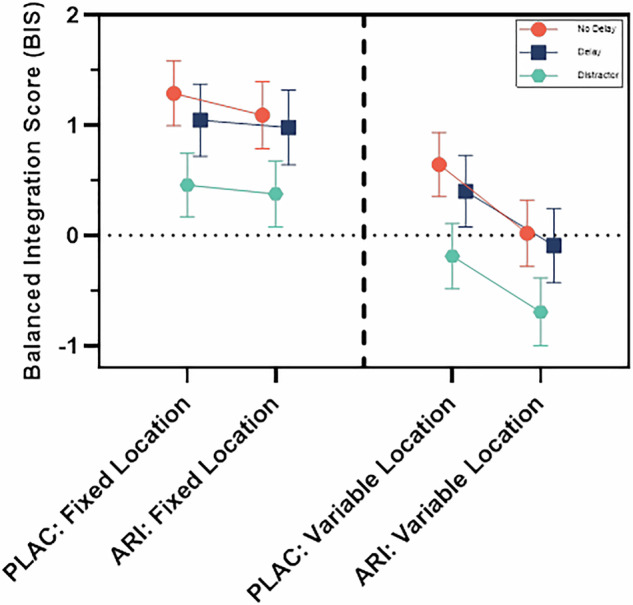
Balanced Integration Score (BIS) data from visuospatial working memory task, comparing placebo (PLAC) to aripiprazole (ARI) in fixed location control trials (left) and variable location trials (right), with separate coloured lines for delay conditions. The BIS measures accuracy adjusted for reaction time; lower scores indicate poorer performance. Values are estimated marginal means ± SEM from generalised linear mixed model. The main effect of treatment condition showed that aripiprazole led to poorer performance compared to placebo overall. Analysis of task effects showed poorer performance in variable location trials compared to fixed location control trials, and poorer performance in distractor trials compared to delay and no delay trials. There was a treatment condition * trial difficulty interaction, indicating that this was due to aripiprazole specifically impairing performance in variable location trials and not in fixed location trials. Model r^2^ = 0.67.

Analysis of the main effect of treatment condition showed that aripiprazole significantly impaired performance compared to placebo on the BIS (p = 0.015, F = 3.14, df1 = 4, df2 = 278). Analysis of task effects showed a significant main effect of trial difficulty, with poorer performance in variable location trials compared to fixed location trials (p = 1.93 × 10^−8^, F = 18.95, df1 = 2, df2 = 278) (Fig. 4). There was also a significant main effect of delay type (p = 0.00095, F = 4.78, df1 = 4, df2 = 278). *Post hoc* tests revealed that there was significantly poorer performance in the distractor vs no delay and delay conditions (both FDR corrected p-values = 0.00015), but no difference between the delay and no delay conditions (FDR corrected p-value = 0.44). There was no effect of order (p = 0.20, F = 1.67, df1 = 1, df2 = 278).

There was a significant interaction between treatment condition and trial difficulty (p = 0.049, F = 3.90, df1 = 1, df2 = 278), but no interaction between treatment condition and delay (p = 0.86, F = 0.15, df1 = 2, df2 = 278). The condition*trial difficulty interaction was driven by impairments in performance on aripiprazole in variable location trials (FDR corrected p-value = 0.015), as aripiprazole did not impair performance compared to placebo in fixed location trials (FDR corrected p-value = 0.84).

Having shown that aripiprazole impaired task performance on the BIS, we sought to determine whether this was due to impairments in response accuracy or response latency. We did not find a main effect of treatment condition for VS-WM response accuracy (p = 0.17, F = 1.61, df1 = 4, df2 = 3083), but found a significant main effect of treatment condition for VS-WM response latency (p = 0.022, F = 2.84, df1 = 4, df2 = 3083), with slower responses on aripiprazole compared to placebo (Supplementary Figures S3 & S4). However, we found no effect of aripiprazole compared to placebo on response time to the vowel/consonant distractor during the task (b = −64.76, t(1052) = −0.76, p = 0.45).

As detailed in our previous publication, we found that aripiprazole induced parkinsonian symptoms compared to placebo on the SAS [65]. There was no relationship between the change in parkinsonian symptoms between the baseline assessment and the aripiprazole assessment, or between the placebo assessment and the aripiprazole assessment, and the difference in response latency in all trials across the same time periods (n = 23, rho=0.34, p = 0.12 for aripiprazole vs baseline; n = 24, rho = −0.22, p = 0.31 for aripiprazole vs placebo). We also found no relationship between the change in the BIS or in response latency between the baseline and aripiprazole assessments and plasma aripiprazole levels (BIS: n = 23, r = −0.19, p = 0.38. Response latency: n = 23, r = 0.13, p = 0.54).

### Comparison between effects of amisulpride and aripiprazole on VS-WM

Relative to placebo, amisulpride and aripiprazole did not differ in their effects on the VS-WM BIS (amisulpride b = −0.38 ± SE 0.16; aripiprazole b = −0.33 ± SE 0.11; t(49) = −0.24, p = 0.81), VS-WM response latency (amisulpride b = 162.55 ± SE 33.167; aripiprazole b = 91.68 ± SE 30.53; t(49) = 1.57, p = 0.12), or VS-WM response accuracy (amisulpride b = −1.45 ± SE 2.68; aripiprazole b = 3.30 ± SE 1.87; t(49) = −1.46, p = 0.15).

### Subjective effects of amisulpride and aripiprazole

There was no significant effect of amisulpride vs placebo on the VAS factors of alertness (b = 4.85, t(50) = 1.09, p = 0.28) or tranquillity (b = 1.51, t(50) = 0.45, p = 0.65). There was also no effect of aripiprazole vs placebo on alertness (b = 6.17, t(48) = 1.54, p = 0.13) or tranquillity (b = 1.00, t(48) = 0.35, p = 0.73).

## Discussion

### Summary of main findings

To our knowledge, this is the first study to investigate the effects of repeated antipsychotic administration on WM or response inhibition in healthy humans, whilst having a longer duration and larger sample size than the two prior studies on sustained attention. We found that both sustained administration of a D2/D3 antagonist and sustained administration of a D2/D3 partial agonist impairs VS-WM memory performance in healthy humans. Furthermore, both antipsychotics impaired VS-WM function in the absence of subjective changes in alertness or mood. We also show that antipsychotics do not cause generalised cognitive impairment, by finding no role for dopamine D2/D3 signalling in response inhibition or sustained attention. This study provides causal evidence in humans of the cognitive effects of sustained antipsychotic administration, and indicates the key role of D2/D3 signalling in regulating working memory processes.

Further analysis indicated that the antipsychotic induced impairments in our composite VS-WM outcome measure (accuracy adjusted for reaction time) were due to increased response latency, as VS-WM response accuracy was not altered following either antipsychotic in comparison to placebo. The effect that we observed on response latency was not related to a generalised motor or cognitive impairment, as the drug induced impairments in response time were not correlated with drug induced parkinsonian symptoms. Moreover, in the same subjects, neither drug affected response latency for a vowel-consonant decision within the same task, response latency during a separate task of sustained attention, or response latency during the Monetary Incentive Delay task (reported in our previous publication) [65].

### Clinical implications

The doses of antipsychotics used in our study are clinically effective doses in the treatment of schizophrenia and other disorders [52, 69]. Our findings are therefore relevant for the tens of million people worldwide who take long-term antipsychotic drugs each year [27, 28]. Although impairments in VS-WM performance are evident in people with schizophrenia and other neuropsychiatric disorders not taking antipsychotic medications, they indicate that sustained antipsychotic treatment with either D2/D3 antagonists or partial agonists may worsen these deficits. Our findings of increased response latency following sustained antipsychotic treatment correspond well with reports by people who take long term antipsychotic treatment of subjective cognitive “slowing” (although we did not find subjective effects on the VAS factors of alertness or tranquillity) [23]. In contrast, given that we found no effect of either amisulpride or aripiprazole on the SART, they suggest that the large effect size deficits in response inhibition and sustained attention tasks including the SART in people with schizophrenia and other disorders are not solely attributable to them taking D2/D3 antagonists or partial agonists, but instead suggest that they may arise from the underlying disease process or an interaction between the disease process and antipsychotic treatment [70].

### Nature of antipsychotic induced changes in VS-WM and comparison with previous findings

Our finding that sustained D2/D3 antagonism or D2/D3 partial agonism did not alter visuospatial working memory performance accuracy, taken together with the impairments in response latency and in the Balanced Integration Score (accuracy adjusted for reaction time) induced by both drugs compared to placebo in the VS-WM task indicates that participants responded more slowly in order to maintain accurate responses following D2/D3 antagonism and D2/D3 partial agonism. This evidence of D2/D3 modulation of speed-accuracy trade-offs extends previous findings that acute D2/D3 modulation alters speed-accuracy trade-offs in healthy humans to provide causal evidence that this effect persists with sub-chronic D2.D3 antagonism and partial agonism [71]. Whether people with schizophrenia and related disorders also alter speed-accuracy trade-offs in order to maintain visuospatial working memory performance accuracy following antipsychotic treatment warrants further investigation, as the inability to effectively deploy this strategy would explain some of the deficits seen in clinical populations in WM performance accuracy.

In sum, this evidence of altered speed-accuracy trade-offs, combined with slower responses which did not appear to be related to a generalised motor impairments may suggest a deficit in WM retrieval following sub-chronic D2/D3 receptor antagonism or partial agonism. WM retrieval has been proposed to be a decision process, in which the decision threshold is regulated by dopaminergic signaling [72–74]. This interpretation is supported by studies in rodents showing that increases in dopamine lower decision thresholds, whilst D2/D3 antagonism increases decision thresholds [71, 75, 76]. In humans, acute administration of L-Dopa, the precursor to dopamine, has been shown to reduce decision thresholds [71]. Our data in humans following repeated D2/D3 antagonism or partial agonism are consistent with the interpretation that impairing D2/D3 signalling increases decision thresholds, which future research could test directly using computational models.

In terms of effect sizes, we did not find evidence to support the hypothesis that D2/D3 partial agonists are less cognitively impairing in comparison to full antagonists [46, 50]. In contrast to this hypothesis, we found that the effect size between amisulpride-placebo and aripiprazole-placebo did not differ on any VS-WM or SART outcome measure, indicating that the magnitude of impairment induced by D2/D3 partial agonism was similar to that induced by D2/D3 antagonism. However, in exploratory analysis on the interaction between VS-WM trial difficulty and treatment condition, we found that aripiprazole impaired performance only in the more difficult variable location trials in terms of accuracy adjusted for reaction time (BIS), whereas the D2/D3 antagonist amisulpride caused impairments on this measure in both fixed location and variable location trials. We interpret this difference as potentially due to the partial agonism of aripiprazole, as more challenging WM tasks may be more dependent on phasic dopamine release, and animal studies show that D2/D3 partial agonists suppress phasic dopamine signalling more than tonic signalling, whereas antagonists supress both equally [77–80].

In exploratory analyses on the interaction between delay type and treatment condition, we did not find evidence of reduced distractibility following sustained D2/D3 antagonism, in contrast to previous work that demonstrated this following acute administration of sulpiride to healthy humans [36]. In our primary composite outcome measure (BIS), we found that there was no interaction between treatment condition and delay type for either amisulpride or aripiprazole. In further analysis of secondary outcome measures, we found that sustained amisulpride administration actually slowed responses compared to placebo in trials with distraction and in trials with no delay, but not in trials following a delay. Examining the BIS measure suggests that this was again due to speed-accuracy trade-offs, as on this measure we found poorer performance with increasing delays, suggesting that differential speed accuracy trade-offs in delay conditions explain the finding of maintained response latency in delay trials in the amisulpride sample. In contrast to amisulpride, aripiprazole impaired response times overall, with no significant interaction between aripiprazole treatment and delay condition. We lack a robust explanation for these exploratory findings, given that we did not find evidence for this interaction in our primary outcome measure, but note the possibility of a type 1 error as multiple statistical tests were conducted across the study.

### Interpretation of SART data and comparison with previous findings

Contrary to our predictions, we found no effect of sustained D2/D3 modulation on the SART. The lack of impairments in the SART in terms of errors, reaction time or the BIS contrasts with what we found for WM. However, it has been suggested that striatal dopaminergic signalling adjusts decision thresholds for cognition and action specifically when memory information is required, meaning that the more complex VS-WM task which required recall was sensitive to altered decision thresholds, whereas the SART was not [73].

Our finding that 7 days of antipsychotic treatment did not affect any SART measure contrasts with two prior double-blind studies following 4–5 days of antipsychotic administration, which demonstrated impairments in sustained attention measures [34, 43]. This discrepancy may be explained by differences in the measures used. The SART has been considered a compound measure of response inhibition and sustained attention, as it uses a reversed response paradigm (high Go, low No-Go) compared to the “traditionally formatted tasks” used in prior investigations, which have a high No-Go, low Go response format [34, 43, 81]. Although the SART is more cognitively demanding, these tasks are thought to be more selective for sustained attention [81, 82]. We chose to use the SART for comparability with the literature in neurospychiatric disorders, in which impairments in the SART and other high Go, low No-Go tests have been extensively described [70]. Future work could test directly whether response requirements during sustained attention determine sensitivity to D2/D3 modulation.

Notwithstanding this, the SART is a reliable measure of response inhibition, reflected primarily by the errors of commission outcome [82]. We provide the first evidence to our knowledge that sustained antipsychotic administration does not affect response inhibition, in agreement with studies in animals which suggest this process is more sensitive to serotonergic and noradrenergic neurotransmission [83, 84].

### Limitations and future directions

We acknowledge several limitations. It is unclear to what extent the observed effects are mediated by effects on striatal compared to PFC D2/D3 receptors. We consider that effects via striatal D2/D3 receptors are more likely, as D2/D3 receptor density is highest in the striatum, and the effects of antipsychotics on brain haemodynamics have been shown to scale with the density of receptors [73]. Furthermore, systemic and striatal specific administration of D2/D3 antagonists have been shown to impair VS-WM performance in humans and animals, whereas local PFC administration has not [85, 86]. Nevertheless, the localisation of effects is not relevant to the main aim of the study, which was to provide clear causal inferences on the link between sustained antipsychotic administration and cognitive impairment.

It has been proposed that dopamine has an inverted U-shaped relationship with cognitive performance, meaning that both excessive and insufficient dopamine would lead to impairments in cognitive performance and that the effects of dopamine manipulations therefore show baseline dependency [87, 88]. We did not measure baseline dopamine function, but used a within-subject design and included baseline task performance as a covariate in our statistical model in order to account for baseline dependency. A further consideration is the extent to which findings in healthy volunteers are generalisable to clinical populations. Dopamine D2/D3 occupancy is similar in healthy volunteers to people with schizophrenia following repeated antipsychotic administration [69]. However, whether adaptations to repeated antipsychotic administration occur, and the mechanisms underlying this are unknown in healthy humans and in people with schizophrenia [89]. Clarifying this may help to predict or ameliorate adverse effects caused by repeated antipsychotic treatment. In addition, we administered amisulpride and aripiprazole for 7 days each, extending previous double-blind investigations in healthy volunteers on the impact of antipsychotics on WM which have all been limited to single administrations [29, 35–41]. It is however important to recognise antipsychotic treatment in schizophrenia is usually long term, and it is possible that there is tachyphylaxis of antipsychotic effects on WM over the long term. Whilst longer term studies in healthy volunteers would be needed to test this, there is some evidence for decline in WM in schizophrenia [90]. Nevertheless, we acknowledge that an inadequate duration of treatment may have contributed to some of our negative findings, such as the lack of subjective effects.

Aripiprazole binds to 5-HT1a and 5-HT2a receptors, 5-HT2c, histamine-1 and alpha-1 receptors in addition to its partial agonist activity at D2/D3 receptors [69, 91]. However, its affinity for these other receptors is 4-5-fold lower than its affinity for D2/D3 receptors, and receptor occupancy following repeated dosing is 30–70% lower, indicating that although we can’t exclude the involvement of these other receptors, effects are likely to be predominantly mediated via D2/D3 receptors [69, 91]. A recent data driven taxonomy suggests that antipsychotics cluster into four groups [92]. We investigated the cognitive effects of agents representative of the selective dopamine antagonist and dopamine partial agonist clusters, meaning that our results might not be generalisable to antipsychotics which were characterised as primarily dopaminergic/serotonergic antagonists or dopaminergic/muscarinic antagonists. We chose these agents as the mechanism of D2/D3 blockade is common to all antipsychotics used over the past 70 years, whilst also including a dopamine partial agonist to test the hypothesis that they are less cognitively impairing [46, 50]. Additionally, equivalent doses of antipsychotics are imprecise and 400 mg of amisulpride has at times been considered to be more equivalent to 15 mg of aripiprazole than to 10 mg [52]. Despite this, the effects we found of the two drugs on cognitive function were remarkably similar, and although plasma levels of both antipsychotics were variable between subjects, we did not find significant relationships between antipsychotic plasma levels and changes in our VS-WM outcome measures. Future research could test whether antipsychotics which also modulate other neurotransmitter systems differ in their impacts on cognitive function, in addition to testing dose dependent effects following repeated administration, and the extent to which adaptations occur over time to the cognitive effects of antipsychotics.

### Summary

In conclusion, we provide the first causative evidence in healthy humans that visuospatial working memory function is impaired following sustained antagonism or partial agonism of D2/D3 receptors by antipsychotics. We found that response accuracy during working memory was maintained at the cost of increased response latency. Contrary to predictions, we found that sustained D2/D3 partial agonism caused working memory impairments of a similar magnitude to D2/D3 antagonism. Our findings are not suggestive of a generalised cognitive effect, as we found no role for D2/D3 signalling in regulating response inhibition or sustained attention, but we demonstrate a key role for D2/D3 signalling in working memory.

## Supplementary information

Supplementary material

## Data Availability

The conditions of the ethical approval of this study do not permit unrestricted access to the raw data. De-identified individual participant data are available for research purposes from the corresponding author (martin.osugo@kcl.ac.uk) from the publication date, subject to a data-sharing agreement, with the exception of data from a minority of subjects who did not consent to de-identified data being used to support future research. Requests will be responded to within 15 working days. The conditions of the ethical approval of the study stipulate that access to data which may allow identification of volunteers will only be permitted for research that has been independently reviewed by an ethics committee.

## References

[1] Ott T, Nieder A. Dopamine and cognitive control in prefrontal cortex. Trends Cogn Sci. 2019;23:213–34. 30711326 10.1016/j.tics.2018.12.006

[2] Beste C, Willemssen R, Saft C, Falkenstein M. Response inhibition subprocesses and dopaminergic pathways: Basal ganglia disease effects. Neuropsychologia. 2010;48:366–73. 19782093 10.1016/j.neuropsychologia.2009.09.023

[3] Brozoski TJ, Brown RM, Rosvold HE, Goldman PS. Cognitive deficit caused by regional depletion of dopamine in prefrontal cortex of rhesus monkey. Science. 1979;205:929–32. 112679 10.1126/science.112679

[4] Phillips AG, Ahn S, Floresco SB. Magnitude of dopamine release in medial prefrontal cortex predicts accuracy of memory on a delayed response task. J Neurosci. 2004;24:547–53. 14724255 10.1523/JNEUROSCI.4653-03.2004 PMC6729988

[5] Watanabe M, Kodama T, Hikosaka K. Increase of extracellular dopamine in primate prefrontal cortex during a working memory task. J Neurophysiol. 1997;78:2795–8. 9356427 10.1152/jn.1997.78.5.2795

[6] Marshall CA, Brodnik ZD, Mortensen OV, Reith MEA, Shumsky JS, Waterhouse BD, et al. Selective activation of Dopamine D3 receptors and norepinephrine transporter blockade enhances sustained attention. Neuropharmacology. 2019;148:178–88. 30633928 10.1016/j.neuropharm.2019.01.003 PMC6424628

[7] Wulaer B, Kunisawa K, Tanabe M, Yanagawa A, Saito K, Mouri A, et al. Pharmacological blockade of dopamine D1- or D2-receptor in the prefrontal cortex induces attentional impairment in the object-based attention test through different neuronal circuits in mice. Molecular Brain. 2021;14:43. 33640003 10.1186/s13041-021-00760-3 PMC7916264

[8] Glickstein SB, Hof PR, Schmauss C. Mice lacking dopamine D2 and D3 receptors have spatial working memory deficits. J Neurosci. 2002;22:5619–29. 12097513 10.1523/JNEUROSCI.22-13-05619.2002 PMC6758242

[9] Linden J, James AS, McDaniel C, Jentsch JD. Dopamine D2 receptors in dopaminergic neurons modulate performance in a reversal learning task in mice. eneuro. 2018;5:ENEURO.0229-17.2018. 10.1523/ENEURO.0229-17.2018 PMC5844058 29527566

[10] Aalto S, Brück A, Laine M, Någren K, Rinne JO. Frontal and temporal dopamine release during working memory and attention tasks in healthy humans: a positron emission tomography study using the high-affinity dopamine D2 receptor ligand [11C]FLB 457. J Neurosci. 2005;25:2471–7. 15758155 10.1523/JNEUROSCI.2097-04.2005 PMC6725173

[11] Ghahremani DG, Lee B, Robertson CL, Tabibnia G, Morgan AT, De Shetler N, et al. Striatal dopamine D_2_/D_3_ receptors mediate response inhibition and related activity in frontostriatal neural circuitry in humans. J Neurosci. 2012;32:7316–24. 22623677 10.1523/JNEUROSCI.4284-11.2012 PMC3517177

[12] Egerton A, Mehta MA, Montgomery AJ, Lappin JM, Howes OD, Reeves SJ, et al. The dopaminergic basis of human behaviors: a review of molecular imaging studies. Neurosci Biobehav Rev. 2009;33:1109–32. 19481108 10.1016/j.neubiorev.2009.05.005 PMC3797507

[13] Pavese N, Andrews TC, Brooks DJ, Ho AK, Rosser AE, Barker RA, et al. Progressive striatal and cortical dopamine receptor dysfunction in Huntington’s disease: a PET study. Brain. 2003;126:1127–35. 12690052 10.1093/brain/awg119

[14] Williams JC, Zheng ZJ, Tubiolo PN, Luceno JR, Gil RB, Girgis RR, et al. Medial prefrontal cortex dysfunction mediates working memory deficits in patients with schizophrenia. Biol Psychiatry Glob Open Sci. 2023;3:990–1002. 37881571 10.1016/j.bpsgos.2022.10.003 PMC10593895

[15] Rao N, Northoff G, Tagore A, Rusjan P, Kenk M, Wilson A, et al. Impaired prefrontal cortical dopamine release in schizophrenia during a cognitive task: a [11C]FLB 457 positron emission tomography study. Schizophr Bull. 2019;45:670–9. 29878197 10.1093/schbul/sby076 PMC6483585

[16] Kaasinen V, Rinne JO. Functional imaging studies of dopamine system and cognition in normal aging and Parkinson’s disease. Neurosci Biobehav Rev. 2002;26:785–93. 12470690 10.1016/s0149-7634(02)00065-9

[17] Sawamoto N, Piccini P, Hotton G, Pavese N, Thielemans K, Brooks DJ. Cognitive deficits and striato-frontal dopamine release in Parkinson’s disease. Brain. 2008;131:1294–302. 18362097 10.1093/brain/awn054

[18] Lobo MC, Whitehurst TS, Kaar SJ, Howes OD. New and emerging treatments for schizophrenia: a narrative review of their pharmacology, efficacy and side effect profile relative to established antipsychotics. Neurosci Biobehav Rev. 2022;132:324–61. 34838528 10.1016/j.neubiorev.2021.11.032 PMC7616977

[19] Kaul I, Sawchak S, Correll CU, Kakar R, Breier A, Zhu H, et al. Efficacy and safety of the muscarinic receptor agonist KarXT (xanomeline-trospium) in schizophrenia (EMERGENT-2) in the USA: results from a randomised, double-blind, placebo-controlled, flexible-dose phase 3 trial. Lancet. 2024;403:160–70. 38104575 10.1016/S0140-6736(23)02190-6

[20] Reilly JL, Harris MSH, Keshavan MS, Sweeney JA. Adverse effects of risperidone on spatial working memory in first-episode schizophrenia. Arch Gen Psychiatry. 2006;63:1189–97. 17088499 10.1001/archpsyc.63.11.1189

[21] Sakurai H, Bies RR, Stroup ST, Keefe RSE, Rajji TK, Suzuki T, et al. Dopamine D2 receptor occupancy and cognition in schizophrenia: analysis of the CATIE data. Schizophrenia Bulletin. 2012;39:564–74. 22290266 10.1093/schbul/sbr189 PMC3627781

[22] McShane R, Keene J, Gedling K, Fairburn C, Jacoby R, Hope T. Do neuroleptic drugs hasten cognitive decline in dementia? Prospective study with necropsy follow up. Bmj. 1997;314:266–70. 9022490 10.1136/bmj.314.7076.266 PMC2125727

[23] Thompson J, Stansfeld JL, Cooper RE, Morant N, Crellin NE, Moncrieff J. Experiences of taking neuroleptic medication and impacts on symptoms, sense of self and agency: a systematic review and thematic synthesis of qualitative data. Soc Psychiatry Psychiatr Epidemiol. 2020;55:151–64. 31875238 10.1007/s00127-019-01819-2

[24] Feber L, Peter NL, Chiocchia V, Schneider-Thoma J, Siafis S, Bighelli I, et al. Antipsychotic drugs and cognitive function: a systematic review and network meta-analysis. JAMA Psychiatry. 2025;82:47–56. 10.1001/jamapsychiatry.2024.2890 PMC11581732 39412783

[25] Wolf A, Leucht S, Pajonk F-G. Do antipsychotics lead to cognitive impairment in dementia? A meta-analysis of randomised placebo-controlled trials. Eur Arch Psychiatry Clin Neurosci. 2017;267:187–98. 27530185 10.1007/s00406-016-0723-4

[26] Howes O, Fusar-Poli P, Osugo M. Treating negative symptoms of schizophrenia: current approaches and future perspectives. Br J Psychiatry. 2023;223:332–5. 37272623 10.1192/bjp.2023.57

[27] Dennis JA, Gittner LS, Payne JD, Nugent K. Characteristics of U.S. adults taking prescription antipsychotic medications, National Health and Nutrition Examination Survey 2013-2018. BMC Psychiatry. 2020;20:483. 33004022 10.1186/s12888-020-02895-4 PMC7528276

[28] Hálfdánarson Ó, Zoëga H, Aagaard L, Bernardo M, Brandt L, Fusté AC, et al. International trends in antipsychotic use: a study in 16 countries, 2005–2014. European Neuropsychopharmacology. 2017;27:1064–76. 28755801 10.1016/j.euroneuro.2017.07.001

[29] Frank MJ, O’Reilly RC. A mechanistic account of striatal dopamine function in human cognition: psychopharmacological studies with cabergoline and haloperidol. Behav Neurosci. 2006;120:497–517. 16768602 10.1037/0735-7044.120.3.497

[30] Logemann HNA, Böcker KBE, Deschamps PKH, van Harten PN, Koning J, Kemner C, et al. Haloperidol 2 mg impairs inhibition but not visuospatial attention. Psychopharmacology. 2017;234:235–44. 27747369 10.1007/s00213-016-4454-z

[31] Chung Y-C, Park T-W, Yang J-C, Huang G-B, Zhao T, Oh K-Y, et al. Cognitive effects of a single dose of atypical antipsychotics in healthy volunteers compared with placebo or haloperidol. J Clin Psychopharmacol. 2012;32:778–86. 23131890 10.1097/JCP.0b013e318272d10c

[32] Saeedi H, Remington G, Christensen BK. Impact of haloperidol, a dopamine D2 antagonist, on cognition and mood. Schizophr Res. 2006;85:222–31. 16679001 10.1016/j.schres.2006.03.033

[33] Lynch G, King DJ, Green JF, Byth W, Wilson-Davis K. The effects of haloperidol on visual search, eye movements and psychomotor performance. Psychopharmacology (Berl). 1997;133:233–9. 9361328 10.1007/s002130050396

[34] Ramaekers JG, Louwerens JW, Muntjewerff ND, Milius H, de Bie A, Rosenzweig P, et al. Psychomotor, cognitive, extrapyramidal, and affective functions of healthy volunteers during treatment with an atypical (Amisulpride) and a classic (Haloperidol) antipsychotic. J Clin Psychopharmacol. 1999;19:209–21. 10350027 10.1097/00004714-199906000-00003

[35] Luciana M, Collins PF. Dopaminergic modulation of working memory for spatial but not object cues in normal humans. J Cogn Neurosci. 1997;9:330–47. 23965011 10.1162/jocn.1997.9.3.330

[36] Mehta MA, Manes FF, Magnolfi G, Sahakian BJ, Robbins TW. Impaired set-shifting and dissociable effects on tests of spatial working memory following the dopamine D2 receptor antagonist sulpiride in human volunteers. Psychopharmacology (Berl). 2004;176:331–42. 15114435 10.1007/s00213-004-1899-2

[37] Dodds CM, Clark L, Dove A, Regenthal R, Baumann F, Bullmore E, et al. The dopamine D2 receptor antagonist sulpiride modulates striatal BOLD signal during the manipulation of information in working memory. Psychopharmacology (Berl). 2009;207:35–45. 19672580 10.1007/s00213-009-1634-0 PMC2764850

[38] Naef M, Müller U, Linssen A, Clark L, Robbins TW, Eisenegger C. Effects of dopamine D2/D3 receptor antagonism on human planning and spatial working memory. Transl Psychiatry. 2017;7:e1107. 28440817 10.1038/tp.2017.56 PMC5416697

[39] Barrett SL, Bell R, Watson D, King DJ. Effects of amisulpride, risperidone and chlorpromazine on auditory and visual latent inhibition, prepulse inhibition, executive function and eye movements in healthy volunteers. J Psychopharmacol. 2004;18:156–72. 15260903 10.1177/0269881104042614

[40] McCartan D, Bell R, Green JF, Campbell C, Trimble K, Pickering A, et al. The differential effects of chlorpromazine and haloperidol on latent inhibition in healthy volunteers. J Psychopharmacol. 2001;15:96–104. 11448094 10.1177/026988110101500211

[41] Mehta MA, Sahakian BJ, McKenna PJ, Robbins TW. Systemic sulpiride in young adult volunteers simulates the profile of cognitive deficits in Parkinson’s disease. Psychopharmacology (Berl). 1999;146:162–74. 10525751 10.1007/s002130051102

[42] Poddar I, Callahan PM, Hernandez CM, Pillai A, Yang X, Bartlett MG, et al. Oral quetiapine treatment results in time-dependent alterations of recognition memory and brain-derived neurotrophic factor-related signaling molecules in the hippocampus of rats. Pharmacol Biochem Behav. 2020;197:172999. 32702397 10.1016/j.pbb.2020.172999

[43] Beuzen JN, Taylor N, Wesnes K, Wood A. A comparison of the effects of olanzapine, haloperidol and placebo on cognitive and psychomotor functions in healthy elderly volunteers. J Psychopharmacol. 1999;13:152–8. 10475721 10.1177/026988119901300207

[44] Corbin L, Marquer J. Is Sternberg’s memory scanning task really a short-term memory task? Swiss J Psychol. 2013;72:181–96.

[45] Veselinović T, Schorn H, Vernaleken IB, Hiemke C, Zernig G, Gur R, et al. Effects of antipsychotic treatment on cognition in healthy subjects. J Psychopharmacol. 2013;27:374–85. 23118022 10.1177/0269881112466183

[46] Choi HJ, Im SJ, Park HR, Park S, Kim CE, Ryu S. Long-term effects of aripiprazole treatment during adolescence on cognitive function and dopamine D2 receptor expression in neurodevelopmentally normal rats. Clin Psychopharmacol Neurosci. 2019;17:400–8. 31352706 10.9758/cpn.2019.17.3.400 PMC6705103

[47] Luciana M, Depue RA, Arbisi P, Leon A. Facilitation of working memory in humans by a D2 dopamine receptor agonist. J Cogn Neurosci. 1992;4:58–68. 23967857 10.1162/jocn.1992.4.1.58

[48] Mehta MA, Swainson R, Ogilvie AD, Sahakian J, Robbins TW. Improved short-term spatial memory but impaired reversal learning following the dopamine D(2) agonist bromocriptine in human volunteers. Psychopharmacology (Berl). 2001;159:10–20. 11797064 10.1007/s002130100851

[49] Cools R, Sheridan M, Jacobs E, D’Esposito M. Impulsive personality predicts dopamine-dependent changes in frontostriatal activity during component processes of working memory. J Neurosci. 2007;27:5506–14. 17507572 10.1523/JNEUROSCI.0601-07.2007 PMC6672352

[50] Keks N, Hope J, Schwartz D, McLennan H, Copolov D, Meadows G. Comparative tolerability of dopamine D2/3 receptor partial agonists for schizophrenia. CNS Drugs. 2020;34:473–507. 32246399 10.1007/s40263-020-00718-4

[51] Osugo M, Whitehurst T, Shatalina E, Townsend L, O’Brien O, Mak TLA, et al. Dopamine partial agonists and prodopaminergic drugs for schizophrenia: Systematic review and meta-analysis of randomized controlled trials. Neurosci Biobehav Rev. 2022;135:104568. 35131396 10.1016/j.neubiorev.2022.104568

[52] Taylor DM, Barnes TRE, Young AH. The Maudsley Prescribing Guidelines in Psychiatry. Wiley; 2021.

[53] Anwyl-Irvine AL, Massonnié J, Flitton A, Kirkham N, Evershed JK. Gorilla in our midst: An online behavioral experiment builder. Behav Res Methods. 2020;52:388–407. 31016684 10.3758/s13428-019-01237-x PMC7005094

[54] Bond A, Lader M. The use of analogue scales in rating subjective feelings. Br J Med Psychol. 1974;47:211–8.

[55] Robertson IH, Manly T, Andrade J, Baddeley BT, Yiend J. ‘Oops!’: Performance correlates of everyday attentional failures in traumatic brain injured and normal subjects. Neuropsychologia. 1997;35:747–58. 9204482 10.1016/s0028-3932(97)00015-8

[56] Starc M, Murray JD, Santamauro N, Savic A, Diehl C, Cho YT, et al. Schizophrenia is associated with a pattern of spatial working memory deficits consistent with cortical disinhibition. Schizophr Res. 2017;181:107–16. 27745755 10.1016/j.schres.2016.10.011 PMC5901719

[57] Sawaguchi T, Goldman-Rakic PS. D1 dopamine receptors in prefrontal cortex: involvement in working memory. Science. 1991;251:947–50. 1825731 10.1126/science.1825731

[58] Dang JA, Shaw TH, McKnight PE, Helton WS. A closer look at warning cues on the sustained attention to response task performance. Hum Factors. 2022;65:1793–803. 35089114 10.1177/00187208211060708

[59] Liesefeld HR, Janczyk M. Combining speed and accuracy to control for speed-accuracy trade-offs(?). Behav Res Methods. 2019;51:40–60. 30022459 10.3758/s13428-018-1076-x

[60] Lohse K, Kozlowski A, Strube MJ. Model Specification in Mixed-Effects Models: A Focus on Random Effects. Communications in Kinesiology. 2023;1. 10.51224/cik.2023.52.

[61] Bond IG, Machida K, Johnson KA. Daily arousal variation has little effect on sustained attention performance. Curr Psychol. 2024;43:2690–703. 10.1007/s12144-023-04473-9 PMC10022567 37359667

[62] Benjamini Y, Hochberg Y. Controlling the false discovery rate: a practical and powerful approach to multiple testing. J R Stat Soc Ser B (Methodol). 1995;57:289–300.

[63] Wuensch K. Comparing correlation coefficients, slopes, and intercepts. Statistics Lessons. 2007.

[64] Herbert M, Johns MW, Doré C. Factor analysis of analogue scales measuring subjective feelings before and after sleep. Br J Med Psychol. 1976;49:373–9. 990210 10.1111/j.2044-8341.1976.tb02388.x

[65] Osugo M, Wall MB, Selvaggi P, Zahid U, Finelli V, Chapman GE, et al. Striatal dopamine D2/D3 receptor regulation of human reward processing and behaviour. Nat Commun. 2025;16:1852. 39984436 10.1038/s41467-025-56663-7 PMC11845780

[66] Selvaggi P, Osugo M, Zahid U, Dipasquale O, Whitehurst T, Onwordi E, et al. Antipsychotics cause reversible structural brain changes within one week. Neuropsychopharmacology. 2025;50:1275–83. 40335667 10.1038/s41386-025-02120-4 PMC12170900

[67] Zahid U, Osugo M, Selvaggi P, Lythgoe D, Fortunato C, Diederen K, Kiemes A, Wall M, Whitehurst T, Onwordi EC, Statton B, Berry A, Dimitrov M, Lau KYR, McCutcheon R, Murray R, Reis Marques T, Mehta M, Howes O. The effects of dopamine receptor antagonist and partial agonist antipsychotics on the glutamatergic system: a double-blind, randomised, placebo-controlled 1H-MRS cross-over study in healthy volunteers. Br J Psychiatry. 2025. Accepted/In press 10.1192/bjp.2025.10319 40698574

[68] Chapman GE, Osugo M, de Marvao A, Howes OD. Aripiprazole-associated QT prolongation in a healthy study volunteer: a case report and literature review. J Clin Psychopharmacol. 2024;44:591–4. 39442546 10.1097/JCP.0000000000001921

[69] Hart XM, Gründer G, Ansermot N, Conca A, Corruble E, Crettol S, et al. Optimisation of pharmacotherapy in psychiatry through therapeutic drug monitoring, molecular brain imaging and pharmacogenetic tests: Focus on antipsychotics. World J Biol Psychiatry. 2024;25:451–536. 38913780 10.1080/15622975.2024.2366235

[70] Wright L, Lipszyc J, Dupuis A, Thayapararajah SW, Schachar R. Response inhibition and psychopathology: a meta-analysis of go/no-go task performance. J Abnorm Psychol. 2014;123:429–39. 24731074 10.1037/a0036295

[71] Chakroun K, Wiehler A, Wagner B, Mathar D, Ganzer F, van Eimeren T, et al. Dopamine regulates decision thresholds in human reinforcement learning in males. Nat Commun. 2023;14:5369. 37666865 10.1038/s41467-023-41130-y PMC10477234

[72] Pearson B, Raškevičius J, Bays PM, Pertzov Y, Husain M. Working memory retrieval as a decision process. J Vis. 2014;14:2. 24492597 10.1167/14.2.2 PMC3912875

[73] Berke JD. What does dopamine mean? Nat Neurosci. 2018;21:787–93. 29760524 10.1038/s41593-018-0152-y PMC6358212

[74] Perugini A, Ditterich J, Shaikh AG, Knowlton BJ, Basso MA. Paradoxical decision-making: a framework for understanding cognition in Parkinson’s disease. Trends Neurosci. 2018;41:512–25. 29747856 10.1016/j.tins.2018.04.006 PMC6124671

[75] Leventhal DK, Stoetzner C, Abraham R, Pettibone J, DeMarco K, Berke JD. Dissociable effects of dopamine on learning and performance within sensorimotor striatum. Basal Ganglia. 2014;4:43–54. 24949283 10.1016/j.baga.2013.11.001 PMC4058866

[76] De Corte BJ, Wagner LM, Matell MS, Narayanan NS. Striatal dopamine and the temporal control of behavior. Behav Brain Res. 2019;356:375–9. 30213664 10.1016/j.bbr.2018.08.030 PMC6516744

[77] Bäckman L, Waris O, Johansson J, Andersson M, Rinne JO, Alakurtti K, et al. Increased dopamine release after working-memory updating training: Neurochemical correlates of transfer. Sci Rep. 2017;7:7160. 28769095 10.1038/s41598-017-07577-y PMC5540932

[78] Duvarci S, Simpson EH, Schneider G, Kandel ER, Roeper J, Sigurdsson T. Impaired recruitment of dopamine neurons during working memory in mice with striatal D2 receptor overexpression. Nat Commun. 2018;9:2822. 30026489 10.1038/s41467-018-05214-4 PMC6053467

[79] Hamamura T, Harada T. Unique pharmacological profile of aripiprazole as the phasic component buster. Psychopharmacology. 2007;191:741–3. 17205315 10.1007/s00213-006-0654-2

[80] Dahan L, Husum H, Mnie-Filali O, Arnt J, Hertel P, Haddjeri N. Effects of bifeprunox and aripiprazole on rat serotonin and dopamine neuronal activity and anxiolytic behaviour. J Psychopharmacol. 2009;23:177–89. 18515444 10.1177/0269881108089586

[81] Carter L, Russell PN, Helton WS. Target predictability, sustained attention, and response inhibition. Brain Cogn. 2013;82:35–42. 23501702 10.1016/j.bandc.2013.02.002

[82] Stevenson H, Russell PN, Helton WS. Search asymmetry, sustained attention, and response inhibition. Brain Cogn. 2011;77:215–22. 21920656 10.1016/j.bandc.2011.08.007

[83] Eagle DM, Bari A, Robbins TW. The neuropsychopharmacology of action inhibition: cross-species translation of the stop-signal and go/no-go tasks. Psychopharmacology (Berl). 2008;199:439–56. 18542931 10.1007/s00213-008-1127-6

[84] Chamberlain SR, Robbins TW. Noradrenergic modulation of cognition: therapeutic implications. J Psychopharmacol. 2013;27:694–718. 23518815 10.1177/0269881113480988

[85] Khan ZU, Muly EC. Molecular mechanisms of working memory. Behav Brain Res. 2011;219:329–41. 21232555 10.1016/j.bbr.2010.12.039

[86] Floresco SB, Magyar O. Mesocortical dopamine modulation of executive functions: beyond working memory. Psychopharmacology (Berl). 2006;188:567–85. 16670842 10.1007/s00213-006-0404-5

[87] Cools R, D’Esposito M. Inverted-U-shaped dopamine actions on human working memory and cognitive control. Biol Psychiatry. 2011;69:e113–25. 21531388 10.1016/j.biopsych.2011.03.028 PMC3111448

[88] van den Bosch R, Hezemans FH, Määttä JI, Hofmans L, Papadopetraki D, Verkes RJ, et al. Evidence for absence of links between striatal dopamine synthesis capacity and working memory capacity, spontaneous eye-blink rate, and trait impulsivity. Elife. 2023;12:e83161. 37083626 10.7554/eLife.83161 PMC10162803

[89] Zahid U, McCutcheon RA, Borgan F, Jauhar S, Pepper F, Nour MM, et al. The effect of antipsychotics on glutamate levels in the anterior cingulate cortex and clinical response: a (1)H-MRS study in first-episode psychosis patients. Front Psychiatry. 2022;13:967941. 36032237 10.3389/fpsyt.2022.967941 PMC9403834

[90] Fett AJ, Velthorst E, Reichenberg A, Ruggero CJ, Callahan JL, Fochtmann LJ, et al. Long-term changes in cognitive functioning in individuals with psychotic disorders: findings from the suffolk county mental health project. JAMA Psychiatry. 2020;77:387–96. 31825511 10.1001/jamapsychiatry.2019.3993 PMC6990826

[91] Kaar SJ, Natesan S, McCutcheon R, Howes OD. Antipsychotics: mechanisms underlying clinical response and side-effects and novel treatment approaches based on pathophysiology. Neuropharmacology. 2020;172:107704. 31299229 10.1016/j.neuropharm.2019.107704

[92] McCutcheon RA, Harrison PJ, Howes OD, McGuire PK, Taylor DM, Pillinger T. Data-driven taxonomy for antipsychotic medication: a new classification system. Biol Psychiatry. 2023;94:561–8. 37061079 10.1016/j.biopsych.2023.04.004 PMC10914668

