# Supplementary material for: Effects of antipsychotics on human cognitive function: causal evidence from healthy volunteers following sustained D2/D3 antagonism, D2/D3 partial agonism and placebo
Source: Mol Psychiatry. 2025 Jul 19;30(11):5315–25. doi: 10.1038/s41380-025-03116-8 (PMC12532602; doi:10.1038/s41380-025-03116-8)
Supplement: Supplementary file 1 — Supplementary material [file 41380_2025_3116_MOESM1_ESM.docx]

**Supplementary Materials for** **“Effects of antipsychotics on human cognitive function: causal evidence from healthy volunteers following sustained D2/D3 antagonism, D2/D3 partial agonism and placebo”**

**Supplementary Methods**

*Screening and safety procedures*

At the screening visit, subjects underwent a full physical examination including vital signs, psychiatric, medical and medication history, electrocardiogram (ECG), blood sampling (screening bloods: full blood count, liver function, urea and electrolytes, c-reactive protein), urine drug of abuse testing, and urine pregnancy testing (females). All of these procedures were repeated at all five study appointments, although blood was analysed for drug levels instead of the tests detailed above after the screening appointment. For the entire duration of the study, participants were advised to abstain from alcohol and recreational drugs, and females were advised to use effective contraception, with the latter two verified by urine testing as detailed above. Participants were advised to take the medication at the same time every day and were asked to record the time of the last dose.

*Sequence generation and allocation concealment*

Upon enrolment, participants were randomised to treatment order (amisulpride or placebo first in arm 1, aripiprazole or placebo first in arm 2). Allocation of drug order was determined by the Latin Square design such that the planned sequence of treatments was balanced for order. The study clinicians and subjects were blind to the treatment order, as an independent researcher allocated subject IDs.

Amisulpride/aripiprazole were encapsulated and lactose powder was used to produce placebo capsules which were identical in shape, size, colour and number to the capsules containing active drug. All capsules were dispensed in identical pill bottles.

*Mixed effect model codes*

The code for the analysis of all SART outcomes was as follows:

fitglme(Data, 'Response~DrugCondition + TreatmentOrder + Response_at_Baseline + (DrugCondition|subjectID)', ‘FitMethod’, ‘REMPL’).

The code for the primary analysis of the VS-WM task, the BIS was as follows:

fitglme(Data, BIS~DrugCondition + TreatmentOrder + BIS_at_Baseline + DrugCondition*Delay + DrugCondition*TrialDifficulty+(DrugCondition*Delay|subjectID)+(DrugCondition*TrialDifficulty|subjectID)', 'FitMethod', 'REMPL')

For analysis of RT and accuracy data in the VS-WM task, we also included random intercepts for each participant, and each of the 49 stimuli, and by-participant random slopes for the effect of treatment condition, delay type, and trial difficulty and the interaction between them(1). This was not possible for the BIS as this variable is computed across mean RTs and accuracies per condition/subject, and therefore there were no pseudo-replicates for the interaction of treatment condition*delay*trial difficulty, and we could not include random slopes at the stimuli level. The code was as follows for the analysis of response latency and accuracy:

fitglme(Data, 'Response~DrugCondition + TreatmentOrder + Response_at_Baseline + DrugCondition*Delay + DrugCondition*TrialDifficulty + (DrugCondition*Delay*TrialDifficulty|subjectID) + 1|Stimuli)', 'FitMethod', 'REMPL')

The code for the linear mixed effect model analysing the VAS factors was as follows:

fitlme(Data, 'Response~DrugCondition + TreatmentOrder + Response_at_Baseline + (DrugCondition|subjectID)').

The code for the linear mixed effect model analysing RT data from the vowel/consonant distractor was as follows:

fitlme(Data, 'Response~DrugCondition + TreatmentOrder + Response_at_Baseline + (DrugCondition|subjectID)').

The code for the linear mixed effect model for the BIS from the VS-WM task excluding interaction terms was as follows:

fitglme(Data, BIS~DrugCondition + TreatmentOrder + BIS_at_Baseline + Delay + TrialDifficulty + (DrugCondition|subjectID)+(TrialDifficulty|subjectID) + (Delay|SubjectID))', 'FitMethod', 'REMPL')

The code for the linear mixed effect model for accuracy/RT data from the VS-WM task excluding interaction terms was as follows:

fitglme(Data, 'Response~DrugCondition + TreatmentOrder + Response_at_Baseline + Delay + TrialDifficulty+ (DrugCondition|subjectID) + (TrialDifficulty|subjectID) + (Delay|SubjectID) + 1|Stimuli))', 'FitMethod', 'REMPL')

**Supplementary Results**

*Exclusion of data for poor performance*

The following sessions were excluded for poor task performance:

- SART AMI: We excluded three sessions for non-response rates >25% (greater than 50 errors of omission). These three subjects performed particularly poorly and did not appear to engage with or understand the task correctly, recording error of omission rates of 34% and 96% on amisulpride, and 100% at baseline. The remaining sessions had an error of omission rate <10%.
- SART ARI: Five sessions were excluded from the aripiprazole sample, due to error of omission rates between 92.5% and 100% (four on aripiprazole, one at baseline). The remaining sessions had error of omission rates <10%.
- VS-WM AMI: We excluded nine sessions for data quality issues. One subject had a 25.76% non-response rate in the amisulpride condition, and a further session from the same subject was excluded due to a 70% non-response rate at baseline. All three sessions were excluded from two subjects who had a non-response rate >66% in all of the sessions. One further subject did not complete the entire task in the amisulpride condition.
- VS-WM ARI: We excluded three sessions for poor response rates, due to 42% and 98% non-response rates at baseline, and a 52% non-response rate on aripiprazole.

*Effect of amisulpride on response latency in VS-WM task*

Analysis of the main effect of treatment condition showed that amisulpride significantly slowed responses compared to placebo across all trials (p= 5.47x10^-7^, F=8.71, df1=4, df2=2917). Analysis of task effects indicated that there was no main effect of trial difficulty on response latency (p=0.52, F=0.65, df1=2, df2=2917). There was a significant main effect of delay type on response latency (p=0.0041, F=3.83, df1=4, df2=2917). Post-hoc tests revealed that this was due to significantly faster responses in the delay condition compared to the no delay (FDR corrected p-value= 0.0029) and distractor conditions (FDR corrected p-value=0.017), with no significant difference between the distractor and no delay conditions (FDR corrected p=0.48).

Whilst we saw a main effect of delay type, there was also a significant interaction between treatment condition and delay (p=0.0008, F=7.17, df1=2, df2=2917) which may account for this, with no difference in response times on amisulpride compared to placebo following an 8 second delay (FDR corrected p-value=0.43), but slower responses on amisulpride in the no delay (FDR corrected p-value=1.30x10^-6^) and distractor conditions (FDR corrected p-value=8.18x10^-6^). There was no interaction between treatment condition and trial difficulty (p=0.43, F=0.63, df1=1, df2=2917). (Figure S1)

Figure S1. Effect of amisulpride vs placebo on response latency in visuospatial working memory task

*Response latency data from visuospatial working memory task, comparing placebo (PLAC) to amisulpride (AMI) in fixed location trials (left) and variable location trials (right), with separate coloured lines for delay conditions. Higher scores indicate poorer performance, values are estimated marginal means ± SEM of response latency in milliseconds from generalised linear mixed model. The main effect of treatment condition showed that amisulpride led to slower responses compared to placebo. Analysis of task effects showed no difference between fixed location control trials and variable location trials, but faster responses in delay trials compared to no delay and distractor trials. A significant interaction between treatment condition and delay indicated that amisulpride slowed responses in no delay and distractor trials, but not in delay trials. Model r^2^=0.37*

*Effect of amisulpride on accuracy in VS-WM task.*

Analysis of the main effect of treatment condition showed no effect of amisulpride compared to placebo in terms of response error (p= 0.95, F=0.17, df1=4, df2=2917). Analysis of task effects found no main effect of trial difficulty on response error (p=0.11, F=2.25, df1=2, df2=2917). However, there was a significant main effect of delay type (p=0.012, F=3.21, df1=4, df2=2917). Post hoc tests revealed that this was due to poorer performance in the distractor condition compared to the no delay condition (FDR corrected p-value=0.0063), with no significant differences between the distractor and delay conditions (FDR corrected p-value=0.44), or the delay and no delay conditions (FDR corrected p=0.10).

There was no interaction between treatment condition and delay (p=1.00, F=0.033, df1=2, df2=2917) or treatment condition and trial difficulty (p=0.43, F=0.64, df1=1, df2=2917). (Figure S2)

Figure S2. Effect of amisulpride vs placebo on accuracy in visuospatial working memory task

*Error data from visuospatial working memory task, comparing placebo (PLAC) to amisulpride (AMI) in fixed location trials (left) and variable location trials (right), with separate coloured lines for delay conditions. Higher scores indicate poorer performance, values are estimated marginal means ± SEM from generalised linear mixed model. There was no main effect of amisulpride compared to placebo. Analysis of task effects showed no difference between fixed location control trials and variable location trials, but less accurate responses in distractor trials compared to no delay trials. There were no interactions between treatment condition and delay or trial difficulty. Model r^2^=0.25*

*Effect of aripiprazole on response latency in VS-WM task*

Analysis of the main effect of treatment condition showed that there was a significant main effect of treatment condition on response latency (p=0.023, F=2.84, df1=4, df2=3083), with slower responses on aripiprazole compared to placebo across all trials. Analysis of task effects demonstrated no main effect of trial difficulty on response latency (p=0.76, F=0.28, df1=2, df2=3083). There was, however, a significant main effect of delay type (p=0.036, F=2.57, df1=4, df2=3083). Post hoc tests revealed that this was due to slower responses in the distractor condition compared to the no delay condition (FDR corrected p-value= 0.018), with no significant difference between the distractor and delay conditions (FDR corrected p=0.08) and the delay and no delay conditions (p=0.80).

There was no interaction between treatment condition and trial difficulty (p=0.47, F=0.52, df1=1, df2=3083), and no interaction between treatment condition and delay (p=0.57, F=0.55, df1=2, df2=3083). (Figure S3)

Figure S3. Effect of aripiprazole vs placebo on response latency in visuospatial working memory task

*Response latency data from visuospatial working memory task, comparing placebo (PLAC) to aripiprazole (ARI) in fixed location trials (left) and variable location trials (right), with separate coloured lines for delay conditions. Higher scores indicate poorer performance, values are estimated marginal means ± SEM of response latency in milliseconds from generalised linear mixed model. The main effect of treatment condition showed that aripiprazole slowed responses compared to placebo overall. Analysis of task effects showed no difference between fixed location control trials and variable location trials, but slower responses in distractor trials compared to no delay trials. There was no interaction between treatment condition and trial type or treatment condition and delay. Model r^2^=0.34*

*Effect of aripiprazole on accuracy in VS-WM task*

Analysis of the main effect of treatment condition found no effect of aripiprazole compared to placebo on response error (p= 0.17, F=1.61, df1=4, df2=3083). Analysis of task effects demonstrated a significant main effect of trial difficulty on response error (p=0.0038, F=5.59, df1=2, df2=3083), with less accurate responses in variable location trials compared to fixed location trials. There was also a significant main effect of delay type (p=0.025, F=2.79, df1=4, df2=3083]. Post-hoc tests revealed that this was due to significantly poorer performance in the distractor condition compared to the no delay (FDR corrected p-value=0.0014). There were no differences between the distractor and delay conditions (FDR corrected p-value=0.16), or the delay and no delay conditions (FDR corrected p=0.32).

There was no interaction between treatment condition and delay (p=0.79, F=0.24 df1=2, df2=3083), but there was a significant interaction between treatment condition and trial difficulty (p=0.033, F=4.53, df1=1, df2=3083). Post-hoc testing did not survive correction for multiple comparisons (FDR corrected p-values = 0.76 for aripiprazole vs placebo in control trials, p=0.37 for aripiprazole vs placebo in working memory trials). (Figure S4)

Figure S4. Effect of aripiprazole vs placebo on accuracy in visuospatial working memory task

*Error data from visuospatial working memory task, comparing placebo (PLAC) to aripiprazole (ARI) in fixed location trials (left) and variable location trials (right), with separate coloured lines for delay conditions. Higher scores indicate poorer performance, values are estimated marginal means ± SEM from generalised linear mixed model. There was no main effect of aripiprazole compared to placebo. Analysis of task effects showed less accurate responses in variable location trials compared to fixed location trials, and less accurate responses in distractor trials compared to no delay trials. A significant interaction between treatment condition and trial difficulty was not elucidated further by post hoc tests. Model r^2^=0.16*

*Relationship between aripiprazole total moiety and changes in VS-WM function*

We also did not find a significant relationship between aripiprazole exposure (determined as plasma levels of aripiprazole + de-hydroaripiprazole (its active metabolite)) and changes in VS-WM performance when we included levels of its active metabolite; BIS: n=23, r=-0.15, p=0.50. Response latency: n=23, r=0.14, p=0.52)

Table S1 – Summary of effects of amisulpride or aripiprazole compared to placebo on visuo-spatial working memory outcome measures.

|  | **Reaction Time** | **Accuracy** | **Balanced Integration score** |
| --- | --- | --- | --- |
| **Condition** | Slower on both AMI and ARI vs placebo | No effect of AMI or ARI vs placebo | Poorer on both AMI and ARI vs placebo |
| **Delay** | Effect in both samples | Effect in both samples | Effect in both samples |
| **Trial Difficulty** | No effect in either sample | Effect in ARI sample | Effect in both samples |
| **Treatment Condition * Trial Difficulty** | No effect in either sample | Interaction between treatment condition and trial difficulty in ARI sample only, post hoc tests not surviving multiple comparisons | Impaired performance on ARI in variable location trials, but not in fixed location trials  No effect in AMI sample |
| **Treatment Condition *Delay** | Slower responses on AMI in the no delay and distractor conditions, no effect following an 8 second delay.  No effect of ARI | No effect in either sample | No effect in either sample |

AMI=amisulpride, ARI=aripiprazole.

**Supplementary References**

1. Lohse K, Kozlowski A, Strube M. Model Specification in Mixed-Effects Models: A Focus on Random Effects2022.
